# A systematic review of quantitative studies exploring staff views on antipsychotic use in residents with dementia in care homes

**DOI:** 10.1007/s11096-023-01645-2

**Published:** 2023-09-29

**Authors:** Amna Raza, Hannah Piekarz, Sundus Jawad, Tim Langran, Parastou Donyai

**Affiliations:** 1 Reading School of Pharmacy, University of Reading, Reading, UK; 2 NHS Frimley, King Edward VII Hospital, Windsor, UK; 3 Department of Pharmacy and Forensic Science, King’s College London, London, UK

**Keywords:** Antipsychotic, Care homes, Dementia, Staff, Surveys and questionnaires, Systematic review

## Abstract

**Background:**

Despite significant warnings of adverse effects, antipsychotics continue to be prescribed for managing the behavioural and psychological symptoms of dementia (BPSD) in care homes. Information provided by staff working within care homes is a factor that can influence prescribing decisions in residents with BPSD.

**Aim:**

The review aimed to capture care home staff views towards antipsychotics for residents with BPSD and separately analyse tools utilized in the studies, mapping them onto the theory of planned behaviour (TPB).

**Method:**

A comprehensive literature search published in ten databases was conducted between May and July 2020 and updated in July 2021. Studies published in full with no date restriction were included and quality assessed using CROSS checklist. A thematic framework approach was applied to extract data and study tools which were then mapped onto the TPB.

**Results:**

Fourteen studies (2059 participants) were included. Findings identified four overarching themes: attitudes toward antipsychotics (e.g. antipsychotics as an appropriate strategy and effectiveness); barriers to deprescribing (e.g. lower staff education, lack of resources and time, poor medication reviews); measures implemented (e.g. nonpharmacological interventions, medication reviews); and perceived needs of staff (e.g. need for training, financial or clinical support). Identified tools addressed seven but not all components of TPB namely, behavioural, normative and control beliefs, attitude, perceived behavioural control, intention and behaviour.

**Conclusion:**

The positive attitudes toward antipsychotics, the identified barriers to deprescribing and the existing tools not addressing all components of the TPB provide the impetus for further research.

**Supplementary Information:**

The online version contains supplementary material available at 10.1007/s11096-023-01645-2.

## Impact statements

- A systematic review was needed to assess the perception of staff towards antipsychotics for residents with BPSD in care homes by pooling data from multiple studies using a quantitative method of data collection, as no previous review has analysed these findings.
- Findings highlighted that further research is needed due to the positive attitudes of staff toward antipsychotics, identified barriers to deprescribing, and the inadequacy of existing tools in addressing all components of TPB.

## Introduction

Dementia affects over 55 million people globally and is expected to affect 78 million people by 2030 and 139 million by 2050 [1]. The changes that occur during the course of dementia lead to behavioural and psychological symptoms of dementia (BPSD), which encompass the symptoms of disturbed perception, thought content, mood or behaviour [2], affecting up to 90% of these patients [3, 4]. Antipsychotics are sometimes used to manage BPSD, where patients are at risk of harming themselves or others or experiencing severe distress, but their use in dementia is associated with significant risks, including increased mortality and incidence of stroke.

In 2009, the Banerjee report for the Department of Health highlighted the risks of antipsychotic use and provided evidence that their harmful effects outweigh the benefits for most dementia cases [5]. Regulatory agencies such as the UK Medicines and Health Regulatory Agency (MHRA), the US Food and Drug Administration (FDA), and the European Medicines Agency (EMA) have all issued warnings outlining the significant risks accordingly [6]. Meta-analyses have also confirmed evidence of adverse effects in controlled trials of antipsychotics in dementia [7–12].

Although warnings have been issued against their use, antipsychotics are still prescribed in care homes where staff have a significant influence on their use for BPSD. Understanding staff perceptions towards antipsychotic usage is important for informing policymakers and developing deprescribing interventions [13, 14]. Several studies have explored staff perceptions on antipsychotic use for BPSD [14–20], with a systematic review of qualitative studies summarizing the decision-making process and prescribing behaviours of stakeholders for nursing home residents [21]. Another qualitative systematic review also focused on nurses’ attitudes and views towards antipsychotic use in people with dementia [22].

Despite several standalone quantitative studies that have utilized mainly surveys and questionnaires as their methods of data collection to explore staff beliefs on antipsychotic prescribing for BPSD [23–28], no systematic review has synthesised findings from these studies. A search of secondary databases Cochrane reviews, Joanna Briggs Institute (JBI) and PROSPERO confirmed this gap, justifying a systematic review of survey studies on staff perceptions of antipsychotics for residents with dementia in care homes. Additionally, the stand-alone quantitative studies that were retrieved did not appear to use strong theoretical underpinning, justifying an examination of the actual structure of any studies retrieved through a systematic search. Based on the research group’s previous expertise, a decision was made to compare retrieved studies against the structure of the Theory of Planned Behaviour (TPB).

## Aim

This review aimed to capture views of staff in care homes towards giving antipsychotics to residents with BPSD and to map the data collection tools onto the TPB to identify gaps in TPB domains [29].

## Method

The reporting of the systematic review followed the Preferred Reporting Items for Systematic Reviews and Meta-Analyses (PRISMA) guidelines [30], with the protocol published on PROSPERO (CRD42021256879). The PICOS for the systematic review was defined as follows: (P) Population, staff directly employed by care homes; (I) Intervention, use of antipsychotics for residents with BPSD; (C) Comparison, perceptions about the use of antipsychotics for residents with BPSD; (O) Outcome, not applicable; (S) Study design, cross-sectional surveys.

A comprehensive literature search was conducted between May and July 2020 using 10 electronic databases: Nursing and Allied Health Literature CINAHL (EBSCO), Cochrane Library, PsychINFO, ProQuest, PubMed, Taylor & Francis, Scopus, Web of Science, Wiley Online Library, and ScienceDirect. The search strategy was tailored for each database (supplementary material A & B), with input from an expert librarian. The grey literature was searched through free text searching in journal websites, NICE website, Google Scholar, reference lists of eligible studies and relevant systematic reviews. All retrieved papers from inception to July 2020 were considered for inclusion. The search was updated in July 2021 with the involvement of another reviewer (HP).

### Eligibility criteria

The studies pertinent to the topic and using a quantitative method of data collection were considered for inclusion in the review. Supplementary material C summarises inclusion and exclusion criteria.

### Study selection

Articles from databases were combined and duplicates removed. One reviewer (AR) screened the titles and abstracts and a second reviewer (PD) checked the decision based on eligibility criteria. The full-text screening was conducted by AR and finalised with PD.

### Quality assessment and data extraction

A checklist Consensus-Based Checklist for Reporting of Survey Studies (CROSS) checklist [31] was used to assess the reporting quality of the included studies. This was an amendment to the published protocol. AR and HP carried out this quality assessment independently, and then compared and discussed. Data were extracted from the included papers into Microsoft Excel for analysis.

### Data analysis

Each paper was read in-depth, themes were identified and a thematic framework developed. Using the framework, data relating to themes were extracted from each paper using constant comparison analysis [32], and the findings discussed with PD. Using a similar approach, questionnaire items were read in-depth and mapped onto the components of the TPB.

Briefly, TPB suggests that human behaviour is influenced by behavioural beliefs, normative beliefs, and control beliefs. Behavioural beliefs shape an individual’s attitude towards a particular behaviour by determining beliefs about its outcomes. Normative beliefs influence an individual’s intention to engage in a behaviour by shaping their beliefs about the expectations of others and the social pressure that results from those beliefs. Control beliefs influence an individual’s intention to engage in a behaviour by shaping their beliefs about factors that impact their ability to perform the behaviour. Accordingly, an individual’s intention to engage in a behaviour is a key predictor of whether the behaviour will occur, influenced by attitude towards the behaviour, subjective norm, and perceived behavioural control. Other composite factors, such as outcome evaluation. Motivation to comply and power of control factors can also be considered.

## Results

**Fig. 1:**
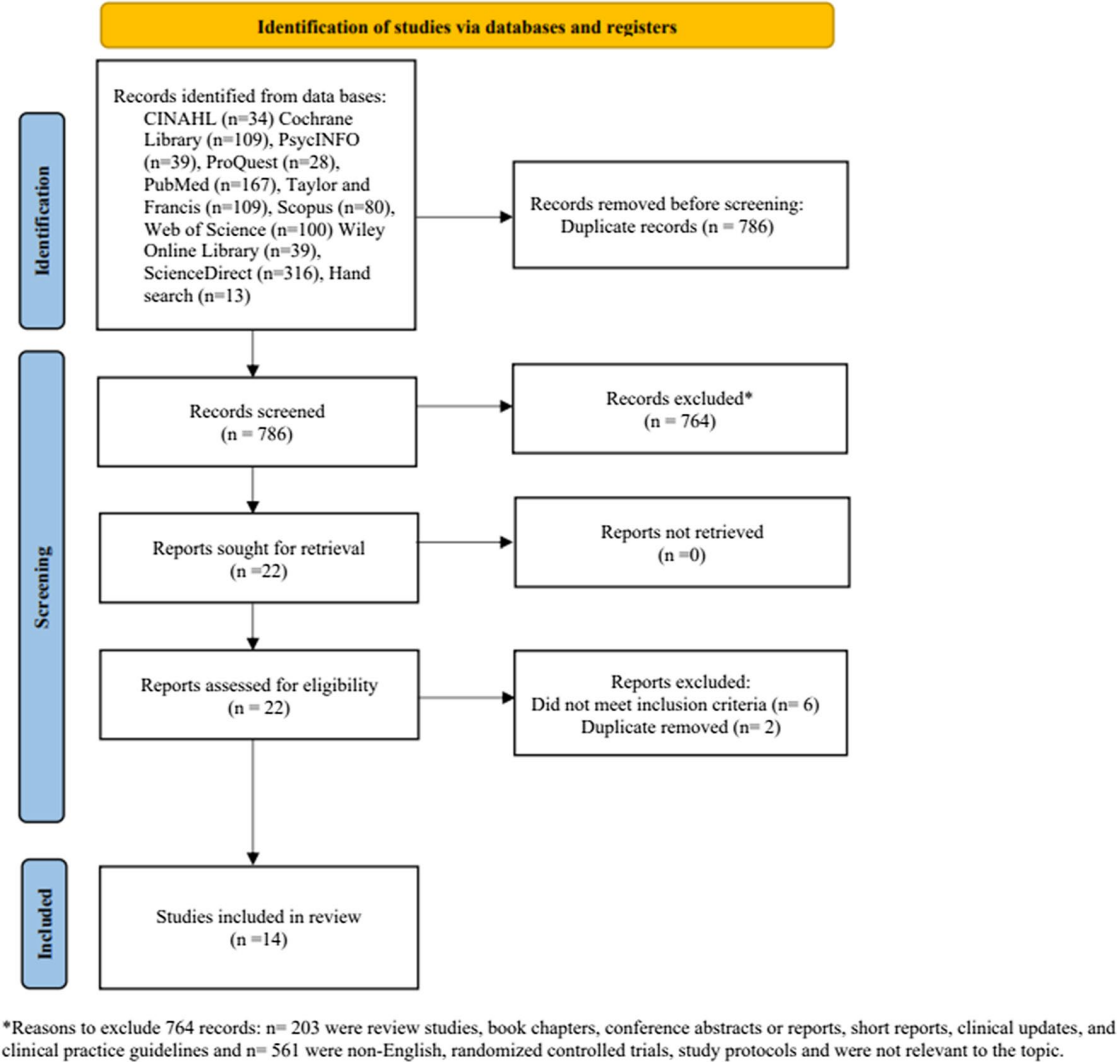
PRISMA flow diagram of included studies

A total of 1034 records were identified from ten databases (n = 1021) and grey literature (n = 13). After reviewing the titles and abstracts, 22 studies were considered for review of full-text articles. Two studies [33, 34] were excluded because they discussed a significant factor already addressed in their earlier paper [35, 36]. Additionally, six other studies [37–42] were excluded as they did not meet the inclusion criteria. After reviewing full texts articles, 14 studies were included in the final review (Fig. 1).

### Quality assessment

Studies were not excluded based on the assessment against the CROSS checklist. Supplementary material D shows whether items of the checklist are reported in the included studies.

### Study characteristics

The included studies (n = 14) were published in the last decade and conducted in the United Kingdom (n = 2) [13, 43], the United States (n = 3) [25, 35, 36], Australia (n = 4) [24, 26, 44, 45], the Netherlands (n = 3) [23, 27, 28], Belgium (n = 1) [46] and New Zealand (n = 1) [47]. Thirteen questionnaires were retrieved from the publications. Studies comprised 2365 participants, including physicians, researchers, pharmacists, patients’ responsible/family caregivers. However, the views of only staff (n = 2059) working in what was described in the studies as nursing homes (n = 7) [23, 25, 27, 28, 36, 45, 46], care homes (n = 2) [13, 43], residential aged care facilities (n = 3) [24, 26, 47], and long-term care facilities (n = 2) [35, 44] were extracted for this review. Table 1 outlines the study characteristics.

**Table 1:** Characteristics of the included studies

Author, year, and country	Participants (n)	Settings	Number of items and method of administration	Method of data analysis	Purpose of the study
Ervin et al., 2012, Australia [24]	Registered nurse, personal care attendants and students or activity coordinators. (n = 130)	Residential aged care facilities	43 items questionnaire and in-person	Descriptive statistics	To determine knowledge, appropriate use, and perceived barriers of pharmacological and nonpharmacological interventions for managing BPSD and resources used to help in managing these behaviours
Cornege-Blokland et al., 2012 [23]	Physicians (n = 27), Nurses (n = 27) and Family Caregivers (n = 32)	Nursing homes	Total number of items not stated and face to face/ telephone	Basic statistics (percentages)	To identify the reasons of antipsychotics prescribing for BPSD and determine the role of nurses and family caregivers which leads to the prescription of antipsychotics in nursing homes
Mavrodaris et al., 2013, UK [13]	GPs (n = 60) and Care home staff (n = 28)	GP Practices and care homes	Total number of items not indicated and, postal and electronic	Thematic analysis	To investigate antipsychotics prescribing practices and patient review at GP practices and in care homes
Lemay et al., 2013, US [25]	Medical Directors (n = 27), Director of nursing (n = 56), Admin (n = 55), Registered nurse (n = 184), Licensed practical nurse (n = 161), Certified nursing assistant (n = 434)	Nursing homes	Total number of items not indicated and postal	Descriptive statistics, chi-squares	To describe nursing staff beliefs and attitudes toward antipsychotics and nonpharmacologic management of dementia-related behaviours, and perceived perceptions for the need of evidence-based training about the safety of antipsychotic medication
Ervin et al., 2014, Australia [26]	Registered nurse, personal care attendants and students or activity coordinators (n = 130)	Residential aged care facilities	43 items questionnaire and in-person	Collaborative analysis and descriptive interpretative approach	Qualitative analysis of staff perceptions of the limitations of pharmacological and nonpharmacological approaches for managing behavioural and psychological symptoms of dementia in residential aged care facilities
Backhouse et al., 2014, England [43]	Care homes managers (n = 299)	Care homes	Total number of items not indicated and postal	Descriptive statistics. Correlations and t-tests	To identify the use of antipsychotics (prevalence of antipsychotics), behaviours and related issues care home staff found difficult to manage and use of nonpharmacological interventions to manage BPSD within care homes
Azermai et al., 2014, Belgium [46]	GPs (n = 28) and Nurses (n = 13)	Nursing homes	Total items not stated and mail	Cross tabulation, correlations and logistic regression model	To investigate the nurses and GP’s attitude towards willingness and barriers to antipsychotic discontinuation in nursing homes
Ellis et al., 2015, Florida USA [36]	Directors of Nursing (n = 109), Nursing home administrator (n = 95), Social worker (n = 7), others n = 65 (managers, nurses, consultants, minimum data set coordinators and other titles)	Conference^a^ (Florida’s Joint Trainings)	19-item survey and in-person	Frequency estimates, descriptive statistics and theme-based content analysis	To assess progress in the current practice implemented to reduce inappropriate antipsychotic medication use in the Florida Nursing Home
Ndukwe et al., 2016, New Zealand [47]	Nurse managers (n = 100)	Residential aged care facilities	22-item survey and mail	Descriptive statistics	Identification of quality use of antipsychotic medicines in residential aged care facilities by reporting specific indicators and contributory factors that influence the antipsychotic use in the aged care facilities
Janus et al., 2017, The Netherlands [28]	Physicians (n = 41), Nurses (n = 81), the patients’ representatives (Proxies) (n = 59)	Nursing homes	16 aspects covered in the questionnaire and e-mail	BWS method (Best-worst scaling approach)	The study aims to determine the attributes and quantification of treatment preferences of physicians, nurses and proxies for challenging behaviours in residents with dementia in nursing homes
Janus et al., 2017, The Netherlands [27]	Nurses and nurse assistant (n = 81)	Nursing home organization	Not stated total number of items and e-mail	Pearson correlation coefficients and linear multiple regression analysis	The study entails the factors (based on the TPB) that influence the nurse and nurse assistant request for antipsychotics for residents with dementia in nursing home
Ludwin et al., 2018, USA [35]	Nurses (n = 158)	Long-term care facilities^b^	Total items not stated and in-person/ advertisements	Descriptive statistics, Multi-Level Modelling	To validate a proposed psychological model to understand treatment choices of care providers in nursing home when managing challenging behaviours in residents with dementia. The model proposed that nurses’ attitudes, self-efficacy, descriptive norms, and outcome expectancies are independently related with nurses’ two treatment choices (antipsychotics and psychosocial) for the management of dementia related behaviours in nursing home residents
Sawan et al., 2019, Australia [45]	Participants (n = 9): Nursing home managers or experienced registered nurse (n = 3), pharmacists (n = 2), Researchers in geriatrics and pharmacotherapy (n = 4)	Nursing homes	68 items questionnaire and e-mail	Content validity Index (CVI)	To develop and content validate a tool that assess organizational culture with respect to the use of psychotropic medicines
Aerts et al., 2019, Australia [44]	Nursing champion (n = 27), GP (n = 22), Person responsible (n = 21)	Long-term care facilities^b^	Number of items not stated and by mail/conducted over the phone	Descriptive statistics and linear mixed models	To identify the reasons for antipsychotics represcriptions and factors responsible for ongoing use of antipsychotics with respect to requests from care staff and perceived behavioural changes

^a^Nursing home professionals attended the conference

^b^Long-term care facilities (include nursing staff)

### Findings of the thematic framework

The thematic framework identified four overarching themes, summarised in Table 2.

**Table 2:** Views about antipsychotics coded into overarching themes and subthemes

Overarching themes	Subthemes
Attitudes toward antipsychotics	Positive beliefs about the effectiveness of antipsychotics in BPSD [23, 25, 27, 28, 35]
	Antipsychotics as an appropriate strategy [24, 28]
	Most challenging behaviours [43, 47]
	Antipsychotics for the management of behavioural problems [23, 43, 44, 46]
	Time as a constraint [24]
	Commonly prescribed antipsychotics [13, 47]
	Adverse effects of antipsychotics [23, 26, 47]
	GPs and pharmacists are held responsible [13, 28]
	Negative beliefs about antipsychotics [24, 26]
Barriers to deprescribing	Lower staff knowledge and education [24–27, 46]
	Lack of resources [13, 24, 26]
	Long working hours/lack of time [36, 46]
	Poor antipsychotic medication reviews [13]
Measures implemented within the settings	Behavioural assessment tools [47]
	Nonpharmacological interventions [36, 43, 44, 47]
	Medication reviews [36, 44, 46, 47]
	Dose adjustment of antipsychotics [36, 46]
	Staff education [36, 47]
Perceived needs of staff	Need for education or guidance [13, 25, 36]
	Training [13, 36]
	Financial resources or clinical support [13, 36]

#### Attitudes toward antipsychotics

Staff generally appeared to hold *positive beliefs about the effectiveness of antipsychotics in BPSD* [23, 25, 27, 28, 35] perceiving a beneficial effect on patients. Staff also viewed the use of *antipsychotics as being an appropriate strategy* in the management of BPSD in case-oriented scenarios [24, 28].

Antipsychotics were primarily used to manage challenging behaviours [23, 44, 46, 47]. The *most challenging behaviours* included aggression [35, 47], agitation [47], sundowning [47], shouting [47], punching [47] and resistance to care [43, 47]. The *most reported challenging behaviours* included agitation [23, 44, 46], aggression [23, 43, 44], activity disturbance [46], and hallucination [46]. *Time* was a major constraint in managing behavioural problems [24]. The *commonly prescribed antipsychotics* measured in one study included risperidone (reported by 83% of respondents), quetiapine (84%), olanzapine (37%), haloperidol (39%) and clozapine (21%) [47] and the alternative to risperidone measured by another study was quetiapine [13].

The most frequently *identified or expected adverse effects* of antipsychotics were increased risk of falls [23, 26, 47], sedation [23, 47], decreased mobility [26], cognitive decline [23], weight loss due to drowsiness [26], weight gain [47] and parkinsonism [23]. *GPs were held responsible* for showing reluctance to review and deprescribe [13], and antipsychotic treatment choice was perceived to be determined by *GPs and pharmacists* [28]. Some staff reported *negative beliefs about antipsychotics* as being less effective than nonpharmacological strategies [24], but these were frequently used for managing BPSD nonetheless [24]. The barriers to administering antipsychotics included adverse effects, resident’s non-compliance, lack of effectiveness and staff education [24, 26].

Overall, attitudes towards antipsychotics for BPSD were largely positive, with assumptions made about their effectiveness, usefulness and relative safety.

#### Barriers to deprescribing

Barriers to reduction or discontinuation of antipsychotics included care home staff *lack of knowledge and education* on appropriate nonpharmacological measures to manage BPSD [24, 26, 27, 46] and *available resources* to support them [13, 24, 26]. Specifically, directors, leaders, and medication administrators had insufficient knowledge regarding the adverse effects [25]. Two studies identified staff *longer working hours or lack of time* as barriers to deprescribing [36, 46]. The other barriers to deprescribing were the negative effects on residents’ quality of life, risk of harm to residents and others, increased staff workload, recurrence of problematic behaviours, and requirement for intensive observation [46]. Some care home staff reported *poor 3-monthly antipsychotic medication reviews*, and only a third of homes reported consistent 6-weekly medication reviews among people initiated on antipsychotics [13].

#### Other measures implemented within settings

Measures were implemented in some care homes to manage BPSD and reduce the use of antipsychotics. These included *behavioural assessment tools* [47], *nonpharmacological interventions* [36, 43, 44, 47], *medication reviews* [36, 44, 46, 47], *dose adjustments* [36, 46] and staff *education* about dementia and its management [47], and psychoactive drugs [36].

#### Perceived needs of staff

Staff required *education and training* to manage residents with BPSD, *financial resources* for additional staff or nonpharmacological alternatives, or *clinical support* from other health professionals to decrease antipsychotic use [13, 36]. They wanted to learn more about medication management and challenging behaviours for residents with dementia [25].

### Analysis of the structures of the tools used to measure views

The questionnaire analysis identified seven TPB-related components.

#### Behavioural beliefs

This component relates to staff beliefs about the consequences of using antipsychotics in BPSD. These include staff positive and negative beliefs about antipsychotic use.

##### Positive beliefs

Three studies [23, 25] evaluated beliefs about the frequency and effectiveness of antipsychotics in managing BPSD, while another study measured the positive effects of antipsychotics on residents and staff [27]. These included the least negative effects [28], effectiveness [28], quick response [28], less burden [28], appropriateness for residents’ behaviours [24, 28], and effects on staff such as less monitoring [28], least amount of effort [28], and feasibility [28, 35]. Staff positive beliefs about using antipsychotics were also compared to psychosocial interventions in some studies [24, 28, 35].

##### Negative beliefs

Three studies [23, 25, 47] examined negative beliefs about antipsychotics, such as increased risk of falls [23, 25, 47], weight gain [25, 47], hypotension [25, 47], cognitive problems [23, 25], extrapyramidal symptoms [23, 25, 47], sedation [25, 47], cardiovascular problems [23, 25] and death [23, 25]. One study focused on participants’ awareness of the limitations of antipsychotic use [13]. A second study explored staff beliefs that reducing antipsychotic use is an indicator of high-quality care and that antipsychotics should be avoided or restricted for residents with BPSD [35]. The third study asked whether initiatives to reduce antipsychotic use were achievable [36].

#### Normative beliefs

Four studies captured descriptive norms (beliefs of what others actually do) regarding antipsychotic prescribing or use in BPSD [13, 35, 43, 47], while five studies captured injunctive norms (perceived approval or disapproval of others) about antipsychotic use or prescription [25, 27, 28, 44, 45]. Most studies used residents [27, 28, 45], residents’ family [27, 28, 44, 45], physicians or GPs or specialists [27, 28, 44, 45] and on-site nursing staff or fellow nurses or nursing assistants [27, 28, 45] as referent individuals. Direct care staff [25], pharmacists [28], psychologists [27] and managers [45] were less commonly referenced in the studies.

#### Control beliefs

##### Facilitators (to deprescribing)

Facilitators for deprescribing or preventing antipsychotics discussed were staff education, environmental influences, and staff skills for nonpharmacological approaches. *Staff education* on dementia, BPSD, antipsychotic use, nonpharmacological strategies, and limitations on antipsychotic use were captured in five studies [13, 24, 36, 44, 47]. *Environmental facilitators* included medication review by GPs or pharmacists [13, 36, 46, 47], the use of nonpharmacological measures [43, 47] and guideline recommendations [28]. *Staff skills, confidence, and expertise* for nonpharmacological measures were measured in four studies [13, 24, 25, 35]. Less reported facilitators included pharmacists’ routine interaction with family or friends [36], pharmacist involvement in interdisciplinary team meetings to enhance patient care [36], availability of resources [28, 36], leaders’ satisfaction with direct-care staff training in managing BPSD [25], leaders and staff interest in learning [25], use of behaviours assessment tools [47], effectiveness of alternative management [13], availability of guidelines and strategies for managing residents with BPSD [13].

##### Barriers (to deprescribing)

Barriers to deprescribing or preventing prescribing of antipsychotics include environmental contexts and resources, staff and environment interactions, and other control beliefs of using antipsychotics. Reported *environmental factors* include inadequate staffing [36, 45], lack of medication reviews [13, 45], time constraints [24, 36, 45], workload [45, 46], and lack of training [13, 24, 25, 36]. Rarely reported factors included reimbursement for increased focused care [36], physical restraint as the only alternative [46], and lack of alternative measures [46]. Negative control beliefs related to *staff interactions with the environment* include opinions of colleagues [24], poor coordination between physicians, nursing homes, assisted living facilities, and hospitals [36], family opposition when antipsychotics are discontinued [46], communication issues on-site staff, managers and GPs [45], the perceived willingness of nurses to discontinue antipsychotics when working in a ward of physically dependent residents [46], GPs relying on the reports of on-site staff and staff interactions with residents and their families [45]. Other control beliefs included the cost of antipsychotics [28], staff difficulty in managing behaviours [43], and the negative effects of antipsychotic discontinuation [46].

#### Attitude toward the behaviour

Four studies measured staff attitudes toward antipsychotic prescription, with three focusing on initiation [13, 23, 27] and one on reduction [36].

#### Perceived behavioural control

This component featured in two studies, with one examining the staff’s need for assistance to reduce antipsychotics [36] and the other exploring staff’s feelings of difficulty, ease, and confidence in asking for antipsychotics for BPSD [27].

#### Intention

Three studies measured intention. One study compared the likelihood of requesting an antipsychotic versus trying psychosocial interventions [35]. Another measured the willingness to discontinue antipsychotics for residents with challenging symptoms [46]. The third estimated the tendency and consideration of staff to ask a physician for antipsychotics for residents with BPSD [27].

#### Behaviour

Two studies measured behaviour. One study assessed the consideration or performance of antipsychotic dose reduction or discontinuation (deprescribing) for residents with challenging behaviours [46]. The other assessed whether staff requested a physician or nurse specialist to prescribe antipsychotics for residents with BPSD [27].

## Discussion

To our knowledge, this is the first systematic review to synthesis findings from quantitative studies measuring attitudes of care home staff towards antipsychotic use in residents with BPSD. The review analyzed data from 13 questionnaires in 14 papers. Findings highlight staff’s positive attitudes towards antipsychotics and barriers to deprescribing, with ongoing need for education, training and financial and clinical support. Questionnaire items assessed against the components of the TPB identified gaps that could be addressed in future work.

### Interpretation and further research

Despite a few negative beliefs, positive attitudes of staff towards antipsychotics use in residents with BPSD were evident, similar to a systematic review of qualitative studies in this field [22]. The positive beliefs were largely identified from study findings in nursing homes and long-term care facilities. Studies have also reported positive beliefs regarding the benefits and effectiveness of antipsychotics [20, 48], which contradicts guidelines [49]. Staff education, lack of resources for nonpharmacological interventions, and limited time were as barriers to deprescribing [13, 24–27, 36, 46]. These are consistent with other studies investigating the deprescribing of antipsychotics in residents with BPSD [50, 51]. Prescribers reported feeling pressured by nursing home staff to prescribe medication, and staff demanding antipsychotics as a result of inadequate resources and staffing levels [18, 52]. Given the positive attitudes towards antipsychotics and the identified barriers to deprescribing, further research should focus on evidence-based educational interventions to promote appropriate requesting and prescribing of antipsychotics for residents with BPSD and to influence the perspectives of care home staff.

Staff also perceived that the deprescribing of antipsychotics requires support such as increased staffing, and financial and clinical input, as noted in previous qualitative studies [19]. Stable staffing levels can improve patient outcomes, while effective deprescribing requires leadership support [19]. Experienced nursing home managers should provide sufficient training and support to demonstrate effective leadership [18]. Although pharmacists have pharmacological expertise, they feel ignored in this field, with decisions being made primarily between GPs and nurses [18]. The pharmacist barriers to deprescribing include inadequate collaboration between community pharmacists and GPs, insufficient access to complete records, and inadequate training [53]. Arguably, therefore, for pharmacists to support deprescribing, it is necessary to enhance communication with other healthcare professionals.

Analysis of the studies in the context of TPB domains is potentially helpful to inform a more comprehensive future cross-sectional survey. Among the eleven studies, various domains were addressed, including behavioural [23–25, 28, 44, 46, 47], normative [25, 28, 43, 44, 47] and control beliefs [24–26, 28, 36, 43, 44, 46, 47], as well as attitudes [13, 23, 36], perceived behavioural control [36], intention [46] and actual behaviour [46]. However, upon in-depth analysis, it became evident that the questionnaires used in these studies lacked a theoretical framework, crucial for systematically measuring behavioural determinant and influencing behaviour.

However, three studies had a theoretical underpinning [27, 35, 45]. The first study used a psychosocial model adapted from the TPB, encompassing behavioral, normative, and control beliefs, as well as intentions [35]. The second study used Schein’s theory of organizational culture and focused on normative and control beliefs [45]. The third study applied TPB, incorporating behavioural beliefs, normative beliefs, attitude, perceived behavioural control, intention and behaviour [27]. No study fully incorporated the TPB domains devised by Ajzen (2006) [29] indicating the need for further work to develop a questionnaire using a systematic approach that encompasses all the components of the TPB model to explore care home staff views on giving antipsychotics to residents with BPSD.

### Strengths and weaknesses

The use of the TPB as a theoretical framework is a strength. This theory provided an understanding of the range of factors addressed or not addressed in the studies associated with the intention of prescribing or deprescribing antipsychotics for residents with BPSD. The literature search was robust, and inclusion/exclusion criteria were carefully applied with involvement from multiple reviewers. Limitations include exclusion of non-English papers, qualitative studies, and perspectives of non care home professionals such as physicians and pharmacists. Some topics such as time constraints, poor medication review, and behavioural assessment tools were rarely explored. Since the original tools used in most studies were not available, the review was based on analysis of the items reported in the papers, except for three studies [27, 43, 44]. Finally, inclusion of two papers from the same study may have led to duplication of results [24, 26].

## Conclusion

Positive beliefs about the effectiveness and appropriateness of antipsychotics and barriers to deprescribing provide the impetus for further research. Providing education, training, and resources to care home staff could potentially decrease antipsychotic use. The questionnaire structures revealed gaps that warrant the development of a comprehensive tool with a strong theoretical underpinning.

### Electronic supplementary material

Below is the link to the electronic supplementary material.

Supplementary Material 1
